# The ideal of equal health revisited: definitions and measures of inequity in health should be better integrated with theories of distributive justice

**DOI:** 10.1186/1475-9276-8-40

**Published:** 2009-11-18

**Authors:** Ole Frithjof Norheim, Yukiko Asada

**Affiliations:** 1 Research group in Global Health, Ethics, Culture and Economics, Department of Public Health and Primary Care & Centre for International Health, University of Bergen, Kalfarveien 31, 5018 Bergen, Norway; 2 Department of Community Health and Epidemiology, Dalhousie University, 5790 University Avenue, Halifax, Nova Scotia, B3H 1V7, Canada

## Abstract

The past decade witnessed great progress in research on health inequities. The most widely cited definition of health inequity is, arguably, the one proposed by Whitehead and Dahlgren: "Health inequalities that are avoidable, unnecessary, and unfair are unjust." We argue that this definition is useful but in need of further clarification because it is not linked to broader theories of justice. We propose an alternative, pluralist notion of fair distribution of health that is compatible with several theories of distributive justice. Our proposed view consists of the weak principle of health equality and the principle of fair trade-offs. The weak principle of health equality offers an alternative definition of health equity to those proposed in the past. It maintains the all-encompassing nature of the popular Whitehead/Dahlgren definition of health equity, and at the same time offers a richer philosophical foundation. This principle states that every person or group should have equal health except when: (a) health equality is only possible by making someone less healthy, or (b) there are technological limitations on further health improvement. In short, health inequalities that are amenable to positive human intervention are unfair. The principle of fair trade-offs states that weak equality of health is morally objectionable if and only if: (c) further reduction of weak inequality leads to unacceptable sacrifices of average or overall health of the population, or (d) further reduction in weak health inequality would result in unacceptable sacrifices of other important goods, such as education, employment, and social security.

## Introduction

The past decade witnessed great progress in research on health inequities. The standard use of terms is now firmly established among health researchers and policy makers. In the words of Kawachi, Subramanian, and Almeida-Filho, "health *inequality *is the generic term used to designate differences, variations, and disparities in the health achievements of individuals and groups," "while health *inequity *refers to those inequalities in health that are deemed to be unfair or stemming from some form of injustice" [1]. Researchers and policy makers can now choose their definition of health inequity from a wide menu of views proposed by many scholars in the past decade [2-7]. Although desired results have not always been achieved, many countries and international organizations have for some time embraced a goal of redressing health inequities by improving the health of populations [8]. The attempt to describe existing health inequities continues, and guidance on how to measure health inequities is now available[9,10] along with novel measures of health inequities [11,12].

Given the explosive growth of interest in health inequities in the past decade, it is interesting that the most widely cited definition of health inequity still is, arguably, the one proposed by Whitehead and Dahlgren in 1991: "Health inequalities that are avoidable, unnecessary and unfair are unjust." [13]. The simplicity and all-encompassing nature of this definition are certainly attractive, but it is rudimentary in the light of recent developments in health inequity research. The Whitehead/Dahlgren definition is useful, but not linked to broader theories of justice [3]. For example, health inequalities that are "avoidable or unnecessary" are, presumably, those we as a society could do something about. But are all avoidable health inequalities arising from, say, small differences in income or educational level unjust? Should all non-health inequalities be eliminated if they are associated with inequalities in health? The definition is silent on such important issues.

Moreover, the Whitehead/Dahlgren definition of health equity requires an upgrade given the widespread agreement on both reducing health inequities and increasing overall population health [3,12,14-18]. While normative political theorists disagree as to whether distributive (i.e., equity) and aggregative (i.e., overall) concerns should be considered simultaneously or separately [19,20], policy makers constantly face trade-offs between improving equity in health and increasing the overall health of a population in real life. For example, preventing cardiovascular disease among inner city minorities may be more difficult and costly than among people with higher income and education. The same amount of effort and resources may achieve higher risk reduction among the well-off than the worse-off [3]. But is further improving the health of the well-off,, thereby increasing inequality in health between the well-off and the worst-off, the best policy option? How much weight should policy makers assign to the worst-off if this requires large sacrifices for the better-off? To offer much needed guidance to policy makers, health researchers need to combine distributive and aggregative concerns in conceptualizing fairness [21,22].

The rapidly developing literature on health inequity measurement [11,12] is rarely linked to the Whitehead/Dahlgren definition, or indeed, to any definition of health inequity. How we measure health inequities should reflect our view on how we conceptualize health inequities [10]. To put it differently, our view on health inequity should be clear and expansive enough to offer a foundation on which various measures of health inequity can be developed, explained, and used.

The aim of this paper is to propose a pluralist framework of fair health distribution that addresses shortcomings in the definitions of health inequity proposed in the past. Our framework consists of two principles: the weak principle of health equality and the principle of fair trade-offs. The weak principle of health equality offers an alternative definition of health inequity. In developing this principle, it is not our intention to review the immensely rich literature on health equity; rather, we defend an alternative view of health equity that is grounded in general theories of justice. The principle of fair trade-offs supplements weak equality in health, and when integrated the two principles represent a comprehensive and more practical understanding of health inequity - what we prefer to call fair health distribution. Trade-off questions require balancing health equity and overall population health as well as balancing concern for health with concern for other goods.

## Equality of what?

Reasoning about distributive justice in health needs to be based on a general and reasonable unit of distribution. The term 'health' needs specification. Following Rawls, Nagel, Sen, Parfit and Daniels, we argue that theories of fair distribution should be concerned with individual people's life prospects [15,23-26]. In the context of health, a person's overall lifetime prospect in terms of longevity and health-related quality of life is therefore a reasonable choice. Following Gakidou, Murray and Frenk, we could define the unit of distribution as health-adjusted life expectancy (HALE) [4,27]. Another alternative would be to use a similar measure of lifetime health (without adopting the *ex-ante *perspective) that includes both a time dimension, measured as life years, and a quality of life dimension, understood in terms of morbidity, pain and functioning. Fortunately, such measures of health-adjusted life years (HALYs) exist, although none of them are perfect, for example, quality-adjusted life-years (QALYs) and disability-adjusted life-years (DALYs) [28,29].

Whatever measure is chosen, it can be linked to more general theories of fair distribution of advantage, such as general economic welfare theory, a Rawlsian theory of justice, or Sen's capability approach. Broome has argued that health is important insofar as it contributes to overall well-being [30]. Daniels has argued that health is important in that it contributes to fair equality of opportunity (whether it is linked to Rawls' theory or more general political conceptions of equal opportunity) [25]. Sen has argued that it would be natural to see health, in his view, "escapable morbidity and premature mortality" as elements in the set of 'capabilities' that egalitarians should be concerned about [15,31].

Daniels' extended notion of equal opportunity is closer to Sen's notion of capability - understood as freedom to pursue his or her objectives - than Rawls' original fair equality of opportunity principle (the original principle was meant to secure access to positional goods such as offices and positions only) [3]. It is also interesting to note that if health is seen as an element of people's freedom "to promote or achieve valuable functionings", health has more in common with positive freedom than with resources or utility. In a pluralist framework, equal distribution of freedoms may be assigned a greater weight than aggregative concerns. Freedom is a good everyone has reason to want insofar as it does not interfere with other people's freedom. This means that how we understand health will also influence our views on how to balance competing concerns.

Without taking a stand on which theory is most appropriate, there is room to link health either to a theory of well-being, to a theory of primary goods, or to the capability approach. We suggest that it is acceptable to see health (measured in its most general form as healthy life expectancy) as one intrinsic element in any general conception of advantage linked to a general theory of justice.

## A defence of the weak principle of health equality

We distinguish between strong and weak equality. The most straightforward view of health equity is strong health equality, where every person or group has equal health. In the normative literature on inequalities in health, however, there is almost unanimous agreement that strong equality of health is an unattainable and unattractive goal and should therefore be avoided or modified. For example, in a recent summary of the normative literature on this issue, Asada concludes that:

"Strict equality for all, however, is not an attractive view for various reasons. For example, it denies personal choice. It would be unrealistically expensive. Moreover, it would be unachievable because some determinants of health are beyond human control. Unlike political liberty, strict equality in health for all would not be a feasible nor agreeable goal." [32]

Brock expresses a similar view in somewhat different words:

" [...] some commitment to equality is a central feature of nearly all theory of justice, with most of the dispute being in what respects should people be equal. However, whatever the relevant arguments, strong objections exist to *a fundamental commitment to equality in outcomes *or conditions, both in general and as the basis for a special concern for the worst off." [33] (emphasis added).

Rejecting strong equality of health, the question then becomes when we can reasonably diverge from strong health equality. Below, we will examine four widely held objections to strong equality of health: (1) the levelling down objection, (2) only those inequalities that are social are unjust, (3) individual responsibility, and (4) the problem of biological or technological limitations. On the basis of the examination of these four objections, we defend the weak principle of health equality as a backbone of our proposed framework of fair health distribution.

### Levelling down objection

Health economists, Culyer and Wagstaff, in a widely cited article, define an equitable distribution of health care as "simply one which gives rise to an equal distribution of health." They immediately add the following qualification: "Of course, this will almost certainly have to be qualified by a side condition that greater equality cannot be achieved by reducing the health of some as a deliberate act of policy" [34]. This qualification is a response to a common objection to strong equality, the levelling down objection. Parfit has argued that egalitarians should not be concerned about strong equality, but rather be concerned with giving priority to the worst off [24]. No one would argue that we should "blind the sighted to equalize health states with the blind".

Daniels defines the goal of "equity in health" partly in response to the levelling down objection:

"One natural way to understand the goal of equity in health--the goal of *health egalitarians*--is to say that we should aim, ultimately, to make all people healthy; that is, to help them to function normally over a normal lifespan. Pursuing equality means "levelling up"-- bringing all those in less than full health to the status of the healthy."[35]

We accept the argument that levelling down is never a good thing, but we hold that equality often is. As we demonstrate in the last section, when weak equality and trade-off considerations are combined properly in a pluralist theory of fair distribution, egalitarians can still promote the value of equality. This argument is grounded on a pluralist moral view [31]. Levelling down will never be judged as a good thing for egalitarians if we adopt a pluralist theory that integrates distributional concerns with overall goodness [22]. Equality is not the only value egalitarians promote, but equality is so important that we should not reject it.

### Only those inequalities that are social are unjust

Arguably, the strongest objection historically to defining health equity as strong equality in health has been that health is a natural good and cannot be redistributed by institutions (such as the health care system or more broadly, a welfare system) in the same way as income. Many scholars think the distinction between naturally and socially created inequalities is of moral importance. Fairness or justice is concerned only about socially created inequalities, not naturally created inequalities.

This distinction goes back to Rawls, who, more than 35 years ago, distinguished social goods from natural goods and suggested that health should be considered as a natural good [23]. Health cannot be distributed in the same way as political rights or income. Although Rawls' ideal theory did not discuss the distribution of health and health care at all (Rawls famously assumed disease and disability away and stipulated that the parties in the original position are fully cooperating members of society over a complete life), the view of health as a natural good has survived up to this day. From these two assumptions combined (that health is a natural good, and that the parties in the original positions have normal capabilities of their complete lifespan), it follows, for instance, that a severe mental disability is not a concern for justice [Attempts at extending or modifying these assumptions in Rawls can be found in Daniels (1985/2007) and Pogge(1989). Daniels does not build upon Rawls' original assumptions].

Does this distinction between social and natural goods indeed hold, and is it morally relevant and useful? We shall not attempt to review the very interesting debate about the correct *interpretation *of Rawls' view on this issue, but rather question the factual premise this debate presupposes [36]. In our view, this distinction is irrelevant in thinking about when inequalities in health are unjust.

First, health is primarily a social good. In the world we live in today, the basic institutions of society determine to a large extent the level and distribution of health. According to statistics from the World Mortality Report, life expectancy in many countries has increased by as much as ten years since the early 1970s [37]. This change is mediated through social factors such as economic growth, technology, reduced inequalities, knowledge and investment in public health and health systems. The WHO reports that life expectancy at birth ranges from 77 (for males) and 82 (for females) in Norway to 41 (for both males and females) in Malawi [38]. Natural factors probably play a minor role in explaining this difference. The health of peoples, or nations, is not something given but fundamentally shaped by how societies are organized and how the benefits of cooperation are shared. We know that Malawi is a much poorer country than Norway, and that the social determinants of health (including health care and public health) are unequally distributed between and within the two countries. The literature on the social determinants of health has, convincingly in our view, demonstrated that social factors are dominantly associated with inequalities in health [39,40]. Health is, then, a concern for social justice.

Second, in most cases it is not possible to distinguish between natural and social causes of disease. Diseases, such as cardiovascular disease or cancer, typically result from the complex interaction between genetic and environmental factors (widely understood to include many of the social determinants of health) [41,42]. A person may inherit genes that increase the risk for, say, cardiovascular disease, but this risk is substantially modified by personal behaviour, the environment and culture, and the basic institutional structures in which that person grows up. Singling out one etiological factor as natural and others as social is in practice difficult, if not impossible.

Third, natural and genetic inequalities in health are actually taken seriously in health policy and clinical practice. Convincing arguments are needed to depart from this view. For example, women who have inherited the BRA1 gene that increases their lifetime risk for breast and ovarian cancer by up to 70-80% are typically treated with more concern than others, not less. Indeed, why should genetically inherited disease (caused by the natural lottery) be given less or no priority compared to those who acquire a disease because they live in poverty or lack basic education? Whether risk is associated with unfair social circumstances or is the result of the natural lottery, it affects well-being, opportunities and freedom to the same degree. Disease and risk of disease are not in the same category as the colour of our eyes or beauty in our judgment of social obligations. In clinical practice, no one would consider whether a condition is caused by social or natural factors as a decisive reason for different prioritisation. Practice does not make a thing right, but if we consider principles against well-considered intuitions in reflective equilibrium, this widely held intuition should be considered seriously [43].

Finally, the implications of the distinction between natural and social factors are counterintuitive and not normatively attractive. Some people have low life expectancy because they are poor, lack education and employment. Others may have low life expectancy, even if not so poor, because they happen to be in a natural setting where there are a lot of malaria-carrying mosquitoes. Should this "natural fact" be a factor against a justice concern? "Freedom from malaria" is one of Sen's paradigmatic examples of what an egalitarian theory should focus on [44]. We agree. If anyone thinks that freedom from malaria should not be a concern for justice, it is probably a mistaken expression of the underlying intuitions that there are some health inequalities we cannot, as a society, do anything about. Consider the situation in the early 1980s before the existence of HIV was known, before its ways of transmission was known and before antiretroviral treatment was developed. The fact that some people died prematurely from AIDS at that time could not be considered unfair, because the disease was not possible to prevent or treat.^1 ^Being free from malaria (and HIV today), on the other hand, is a concern for justice because society does have the knowledge and the means to prevent and treat them. In our view, the relevant distinction is whether the institutions of society can respond adequately to a disease or not, which we will elaborate below - not whether the causes are natural or social.

The upshot of this discussion is that most health inequalities should - as a starting point - be considered unjust. The division between health as a natural and a social good is not possible to define. Neither is it morally relevant. [Of course it would be judged unfair if they had been denied access to preventive measures. That many people died prematurely was also a reason to fund HIV research.]

### Individuals should have some responsibility for inequality

Another widely held objection to strong health equality is personal responsibility. Temkin, though his work does not focus specifically on health, proposes the following view of which inequalities are of moral concern: "Egalitarians generally believe that it is bad for some to be worse off than others through no fault or choice of their own" [19]. Sen also argues that the issue of personal responsibility has some bearing on the issue of health inequalities:

"What is particularly serious as an injustice is the lack of opportunity that some may have to achieve good health because of inadequate social arrangements, as opposed to, say, a personal decision not to worry about health in particular. In this sense, an illness that is unprevented and untreated for social reasons (because of, say, poverty or the overwhelming force of a community-based epidemic), rather than out of personal choice (such as smoking or other risky behavior by adults), has a particularly negative relevance to social justice." [15]

Similarly, liberal egalitarian theories of distributive justice argue that a central goal of public policy should be to secure all individuals equal opportunities. All equal opportunity approaches argue that society should eliminate inequalities that arise from factors beyond individual control. One prominent position argues that equal opportunity requires that all inequalities that arise from factors outside the agent's control in the social and the natural lottery, such as a person's natural and genetic abilities should be eliminated, but that inequalities or costs that arise from factors under the agent's control should be accepted [45].

Applied to the context of health the principle of equality implies that all individuals who make the same choices should be treated as if they were identical with respect to all factors outside their own control. This view holds that natural inequalities (associated with, for example, genetic factors) should be a concern for egalitarian justice.

A common misunderstanding of liberal egalitarianism is that these theories argue that individuals should be held responsible for the *consequences *of their choice. In the context of health this would imply that all inequalities in health are counted as fair if the agent in question could have avoided bad health outcomes by making different choices. However, the principle of responsibility states that individuals should be held responsible for their choices, not for the consequences of their choices [46,47]. It is only in the special case where the outcome only depends on the individual's choices and not on any other factors (including the responsibility of society) that this principle implies that individuals should be held responsible for the consequences of their actions. To hold people responsible for the actual consequences of their choice would therefore be to hold them responsible for too much [48]. The implication of the principle of responsibility on the concept of health equality is therefore in practice limited.

Interestingly, health systems of liberal societies generally embody this (correctly understood) principle of responsibility. We discourage people from practising "irresponsible" health behaviours, such as smoking, unsafe sex, and sedentary life styles, through public health and health promotion. For some behaviours, we make people responsible for their action by imposing taxes (e.g., tax on cigarettes) or making them illegal (e.g., seat belt laws). But our health systems do not treat the reckless and the sensible differently.

### Strong equality is unachievable because of limitations of biology and technology

The final objection to strong health equality commonly found in the literature relates to considerations about biological and technological limitations. Many definitions of health inequity proposed by health science researchers suggest that inequalities in health are fair if those inequalities are unavoidable. Whitehead and Dahlgren explicitly incorporate unavoidability in their definition. Similarly, the pragmatic definition of health equity adopted by the International Society for Equity in Health in 2000 focuses on remediability: "Equity in health is the absence of systematic and potentially remediable differences in one or more aspects of health across socially, demographically, or geographically defined populations or population subgroups" [49]. Furthermore, though not as explicit as the two definitions above, Gakidou, Murray, and Frenk, in their proposal for measuring health inequities across countries for *The World Health Report 2000*, consider health inequalities caused by factors amenable to human interventions as unjust [4,27].

The concern for unavoidability in the health equity literature echoes the idea of shortfall equality developed by Sen and Anand for the human development index [50]. They are concerned about the fact that some people are more efficient converters of resources or goods to well-being (or health) than others [51]. Anand and Sen explain:

"In those cases in which human diversity is so powerful that it is impossible to equalize the maximal levels that are potentially achievable, there is a basic ambiguity in assessing achievement, and in judging equality of achievement (or of the freedom to achieve). If the maximal achievement of person 1 -- under the most favourable circumstances -- is, say, x, and that for person 2 is 2x, then equality of attainment would invariably leave person 2 below her potential achievement." [50]

As an alternative to strong or attainment equality, Anand and Sen defend shortfall equality (for a more extended discussion and some reservations, see [16]). This view can most easily be illustrated by reference to gender inequality: There is a commonly observed gender difference in life expectancy of about 2-5 years (researchers disagree about the correct figure), favouring women [52]. In a society where life expectancy is, for example, 60 years for men and women, this equality in life expectancy by sex would be judged equitable if strong equality is the normative standard, while it is inequitable if shortfall equality is the standard.

The key question is: if strong equality is not feasible, should egalitarians be concerned about strong equality or equal shortfall from what is feasible? In short, should we be concerned about all health inequalities (measured from an equal baseline), or only shortfall inequalities (measured from a baseline defined by what is possible)?

We agree with Anand and Sen, that equity concerns inequalities that are avoidable. Although they do not clearly define when we should consider inequalities to be unavoidable, the term often includes limitations of biology, technology or knowledge. Anand and Sen refer to limitations of biology when they defend shortfall equality in the case of men and women as illustrated above. Our view of biological limitations is that, whether they are functional or mental limitations, egalitarians should not count them as legitimate shortfalls. Above, we argued against the view that only those inequalities that are social are unjust. In health policy and clinical practice, we take natural inequalities in health seriously and consider them as important as social inequalities in health. Inequalities due to biology are examples of natural inequalities, and we do not see why gender deserves special consideration among many other biological factors, such as genetics.

It appears reasonable, on the other hand, that egalitarians should be concerned about limitations caused by the level of technology or knowledge available. The implications of this departure from strong equality are probably substantial as technological limitations change over time. To repeat our example from above, using shortfall equality as the standard, people dying prematurely from HIV/AIDS in the early 1980s (before the aetiology of the disease was known) were suffering tremendously, but their tragically reduced life expectancy was not unfair. Given the medical advancement for HIV/AIDS treatment in recent decades, however, the same amount of suffering and premature death now is quite rightly considered inequitable.

We believe the idea of shortfall equality applied to technological limitations reflects a sound principle and well-considered moral intuitions that many people hold regarding equity -although what such limitations entail requires further clarification.

### Statement of the weak principle of health equality

The discussion above on four objections to strong health inequality suggests the following. First, health equity should not be improved by "levelling down," that is, making people less healthy. The objection loses force if concern for equality is integrated with concern for average health as required by a pluralist theory of fair distribution. Second, when considering which health inequalities are unjust, distinguishing social and natural factors is morally irrelevant. Third, health inequalities are acceptable if they are derived completely from choices that free and fully informed adults make. But such health inequalities are extremely rare, and in practice, individuals cannot often be held responsible for health inequalities due to choice. Finally, health inequalities are fair if they are associated with technological limitations on further health improvements.

Taken together, we propose a definition of weak health inequality: every person or group should have equal health except when: (a) health equality is only possible by making someone less healthy, or (b) technological limitations exist to further health improvements. In other words, the weak principle of health equality suggests that health inequalities that are amenable to positive human interventions are unacceptable.

## Trade-off questions

All definitions of health equity proposed in the past have exclusively focused on health inequalities: among many health inequalities, which ones are unfair? The weak principle of health equality is one partial answer to this question. A definition of *fair *distribution of health needs to expand its scope beyond health inequalities. Even if the weak principle of health equality is satisfied - every person or group has equal health adjusted for technological limitations to further health improvements - there may be situations where fairness in distribution rejects weak health inequality.

Achieving health equality is an important goal but is only the first step towards a broader pluralist notion of fair health distribution. The next step involves trade-offs with other objectives. In the world of limited resources, defined broadly in terms of, for example, money, time, and talents, to tackle serious issues in our society, how important is it for us to commit to the weak principle of health equality? We divide such trade-off issues into two categories, trade-off between weak health equality and overall health, and trade-off between health and other goods. Together we call them trade-off questions, which form another backbone of our proposed framework of fair health distribution.

### Trade-off between weak equality and average

Weak equality of health is morally objectionable when further improvement in weak equality leads to *unacceptable *sacrifices of average or overall health of the population. This formulation, of course, leaves open the normative question about which trade-offs between weak equality and average health are unacceptable. It is crucial to note, however, that a framework of fair distribution should acknowledge that weak equality is not the only concern. As noted by Sen, equality is not only a complex notion where there is "internal plurality" within the concept itself. Equality can only be properly understood if it is considered together with other key ethical concepts:

"The demands of equality cannot be clearly interpreted or understood without taking adequate note of efficiency considerations. The point is not merely that the demands of equality have to be ultimately weighted against the force of competing demands, when present. It is also that the interpretation of the demands that equality makes has to be assessed in the light of other considerations (e.g. aggregative concerns) that are *inter alia *recognized. The explicit admittance of other concerns avoids the overburdening of equality with unnecessary loads." [16]

Trade-off questions are important in the context of population-level policies, where the aim is to distribute healthy life years fairly. For example, how much equality of healthy life years between different groups or individuals is a decision-maker is willing to sacrifice, in order to move towards a higher average for all? Or, following Anand: 'what amount of healthy life years, if enjoyed equally by everybody, would have equivalent value to a greater average healthy life expectancy?' [14,53]. Equal health here refers to equality in health that is amenable to positive human intervention.

As yet there is no full-fledged principled account of how balancing of this kind can be handled. But economists have suggested a number of indices that attempt to capture the trade-off between weak equal health (or "equity" as the economists call it) and maximizing concerns (efficiency), including the modified Atkinson's index suggested by Anand [14,53], and the Achievement index proposed by Wagstaff [12], which incorporates concerns, such as (a) aversion to weak inequality of health, (b) a special concern for the worst off individuals or groups, and (c) explicit judgment about the appropriate trade-off between egalitarian concerns (a + b) and maximization concerns [21]. Further investigation is needed to see whether our concerns about fair distributions can be quantified appropriately by these indices. Reasonable quantitative measures coupled with fair processes for making normative choices explicit and legitimate could be used to bridge health policy and the unified framework that we are proposing [54].

### Trade-offs between health and other goods

Weak health equality may also be unfair if further reduction in health inequality would *unacceptably *increase inequality or reduce aggregate well-being in other domains of concern (such as education, employment, social security, and so on). In other words, some departures from weak equality in health are acceptable with reference to other important social objectives. For example, in a welfare state with publicly financed health care, one may justify limits on the provision of high-cost last-chance chemotherapy with marginal health benefits with reference to improvements in social security for the homeless or in primary education. Although many think that health is special, rightly so in our view, health is partly also a tradeable good [55,56].

Health cannot be seen in isolation from other goods, and the interrelation between health and other advantages is of interest theoretically and practically. Weak inequalities in health may be acceptable if we could thereby reduce inequality in other domains more. On the other hand, and this is most often the case, if inequalities in the other domains also increase inequality in health, this may be considered "doubly" unfair. We need a robust theory which integrates health and other substantive goods. Some existing philosophical theories are a good start, as we briefly sketch here. For example, Daniels argues that Rawls' theory of justice provides a unified account of justice that can help explain when social group inequalities in health are unfair: "Health inequalities are unjust or unfair if they result from an unjust distribution of the socially controllable factors that affect health". He suggests that what counts as "an unjust distribution of the socially controllable factors" might follow from Rawls' theory. One problem, however, is that this theoretical framework lacks an index of primary goods that can be used for making trade-offs between health and non-health primary goods.

The Rawlsian framework provides little guidance, Prah Ruger has argued, "when accounts of social and economic justice conflict with accounts of justice with respect to health" [57]. As she points out:

" [B]efore giving substantially greater weight to broader socioeconomic policies than to health policies, we need to understand the precise mechanisms through which various factors influence health. We must then determine how to weight different social objectives, once we have this information. In light of existing information on social determinants of health, it would be unwise to prescribe sweeping policies, such as completely flattening of socioeconomic inequalities, in an effort to improve health."[57]

Sen's capability approach provides an alternative framework for evaluating justice. Sen lists five categories of rights and opportunities that are seen as necessary to help "advance the general capability of a person" [44]. Poverty is defined as capability deprivation in any of these categories. Sen's list includes: political freedoms; economic facilities; social opportunities; transparency guaranties; and protective securities. Health would probably be subsumed under social opportunities, although transparency guarantees and protective guaranties also include relevant aspects of health-related agency and having good health [58]. This framework links health to a general theory of justice in a way that is interesting. Prah Ruger argues that Sen's approach is superior to Rawlsian analysis in this respect [57]. The capability approach, however, requires that we develop a comparable metric of advantage across various domains [31,59,60].

## Concluding remarks

When are inequalities in health unfair? Answers to this question may be useful for people who want to measure inequalities in health. A definition of health equity may also be useful to people who are concerned about priority setting in health and health care.

Our framework of fair health distribution is composed of the weak principle of health equality and the principle of fair trade-offs. The weak principle of health equality offers an alternative definition of health equity to those proposed in the past. The weak principle of health equality maintains the core ideas of the widely popular Whitehead/Dahlgren definition of health equity, and at the same time, it offers a richer philosophical foundation. This principle states that every person or group should have equal health except when: (a) such strong health equality is only possible by making someone less healthy, or (b) technological limitations exist to further health improvements. In short, health inequalities that are amenable to positive human interventions are unacceptable.

The principle of fair trade-offs supplements the weak principle of health equality. The principle of fair trade-offs states that weak equality of health is morally objectionable if and only if: (c) further reductions in weak health inequality leads to unacceptable sacrifices of average or overall health of the population; or (d) further reduction in weak health inequality would result in unacceptable sacrifices of other important goods, such as education, employment, and social security. We believe coupling the weak principle of health equality with the principle of fair trade-offs will offer a fuller view of health equity than the traditional view. A combined pluralist framework is necessary to understand fair health distribution.

## Competing interests

The authors declare that they have no competing interests.

## Authors' contributions

OFN and YA developed the ideas and arguments of the paper together. OFN wrote the first draft. Both authors developed the draft further, read and approved the final manuscript.

## References

[B1] Kawachi I Subramanian SV Almeida-Filho N A glossary for health inequalities J Epidemiol Community Health 2002 56 647 652 10.1136/jech.56.9.647 12177079 PMC1732240

[B2] Braveman P Health disparities and health equity: Concepts and measurement Annual Review of Public Health 2006 27 167 194 10.1146/annurev.publhealth.27.021405.102103 16533114

[B3] Daniels N Just Health 2007 New York: Cambridge University Press

[B4] Gakidou EE Murray CJ Frenk J Defining and measuring health inequality: an approach based on the distribution of health expectancy Bull World Health Organ 2000 78 1 42 54 10686732 PMC2560605

[B5] Hausman DM Asada Y Hedeman T Health inequalities and why they matter Health Care Anal 2002 10 177 91 10.1023/A:1016502119050 12216744

[B6] Marchand S Wikler D Landesman B Class, health and justice Milbank Mem Fund Quarterly 1998 76 3 449 467 10.1111/1468-0009.00098 PMC2751091 9738170

[B7] Starfield B Equity in health Journal of Epidemiology and Community Health 2002 56 483 484 10.1136/jech.56.7.483 12080152 PMC1732189

[B8] Graham H Social determinants and their unequal distribution: Clarifying policy understandings The Milbank Quarterly 2004 82 101 124 10.1111/j.0887-378X.2004.00303.x 15016245 PMC2690205

[B9] Harper S Lynch J Methods for measuring cancer disparities: Using data relevant to Healthy People 2010 cancer-related objectives 2005 79 Bethesda, MD: National Cencer Institute

[B10] Asada Y Health Inequality: Morality and Measurement 2007 Toronto: University of Toronto Press

[B11] Bleichrodt H van Doorslaer E A welfare economics foundation for health inequality measurement Journal of Health Economics 2006 25 945 957 10.1016/j.jhealeco.2006.01.002 16466818

[B12] Wagstaff A Inequality aversion, health inequalities and health achievement J Health Econ 2002 21 4 627 641 10.1016/S0167-6296(02)00006-1 12146594

[B13] Whitehead M The concepts and principles of equity and health Int J Health Serv 1992 22 3 429 445 10.2190/986L-LHQ6-2VTE-YRRN 1644507

[B14] Anand S The concern for equity in health J Epidemiol Community Health 2002 56 485 487 10.1136/jech.56.7.485 12080153 PMC1732197

[B15] Sen A Why health equity? Health Econ 2002 11 659 666 10.1002/hec.762 12457367

[B16] Sen AK Inequality Reexamined 1992 Oxford: Clarendon Press

[B17] Wagstaff A QALYs and the equity-efficiency trade-off Journal of Health Economics 1991 10 21 41 10.1016/0167-6296(91)90015-F 10113661

[B18] Brock D Wikler D Jamison DT, Breman JG, Measham AR, Alleyne G, Claeson M, Evans DB, Jha P, Mills A, Musgrove P Ethical Issues in Resource Allocation, Research, and New Product Development Disease Control Priorities in Developing Countries 2006 Second New York: Oxford University Press and The World Bank 259 270

[B19] Temkin LS Inequality 1993 Oxford: Oxford University Press

[B20] Williams A Cookson R Culyer AJ, Newhouse JP Equity in health Handbook of Health Economics 2000 Amsterdam: Elsevier Science Ltd 1863 1910 10.1016/S1574-0064(00)80048-7

[B21] Norheim OF Implementing the Marmot Commission's Recommendations: Social Justice Requires a Solution to the Equity-Efficiency Trade-Off Public Health Ethics 2009 in press 101093/phe/php00619655049

[B22] Norheim OF A note on Brock: Prioritarianism, egalitarianism and the distribution of life years Journal of Medical Ethics 2009 35 9 565 569 10.1136/jme.2008.028845 19717696

[B23] Rawls J A Theory of Justice 1971 Cambridge, Mass: The Belknap Press of Harvard University Press

[B24] Parfit D Clayton M, Williams A Equality or Priority? The ideal of equality 2002 New York: Palgrave, Macmillan 81 125

[B25] Daniels N Just health care 1985 New York: Cambridge University Press

[B26] Nagel T Equality and partiality 1991 Oxford and New York: Oxford University Press

[B27] Murray CJ Gakidou EE Frenk J Health inequalities and social group differences: what should we measure? Bull World Health Organ 1999 77 7 537 543 10444876 PMC2557698

[B28] Drummond M Stoddart G Torrance G Methods for the economic evaluation of health care programmes 1987 Oxford: Oxford University Press

[B29] Murray CJ Acharya AK Understanding DALYs (disability-adjusted life years) J Health Econ 1997 16 6 703 730 10.1016/S0167-6296(97)00004-0 10176780

[B30] Broome J Qalys Journal of Public Economics 1993 50 149 167 10.1016/0047-2727(93)90047-W

[B31] Sen A The idea of justice 2009 London: Allen Lane/Penguin

[B32] Asada Y Assessment of the health of Americans: the average health-related quality of life and its inequality across individuals and groups Popul Health Metr 2005 3 7 10.1186/1478-7954-3-7 16014174 PMC1192818

[B33] Brock D Rhodes R, Battin M, Silvers A Priority to the Worse Off in health-Care Resource Prioritization Medicine and Social Justice 2002 Oxford: Oxford University Press 362 372

[B34] Culyer AJ Wagstaff A Equity and equality in health and health care J Health Econ 1993 12 4 431 457 10.1016/0167-6296(93)90004-X 10131755

[B35] Daniels N Equity and population health: toward a broader bioethics agenda Hastings Cent Rep 2006 36 4 22 35 10.1353/hcr.2006.0058 16898358

[B36] Pogge TW Realizing Rawls 1989 Ithaca and London: Cornell University Press

[B37] Nations U World mortality report 2005 New York: United Nations

[B38] WHO Working together for health 2006 Geneva: WHO

[B39] Evans RG Barer ML Marmor TR Why are some people healthy and others not?: The determinants of health of populations 1994 New York: Aldine De Gruyter

[B40] World Health Organization, Commission on social determinants of health Closing the gap in a generation : health equity through action on the social determinants of health : final report of the commission on social determinants of health 2008 Geneva: World Health Organization

[B41] Hemminki K Lorenzo Bermejo J Försti A The balance between heritable and environmental aetiology of human disease Nature 2006 7 958 965 10.1038/nrg2009 17139327

[B42] Tiret L Gene-environment interaction: a central concept in multifactorial disease Proceedings of the Nutrition Society 2002 61 457 463 10.1079/PNS2002178 12691175

[B43] Daniels N Wide reflective equilibrium and theory acceptance in ethics The Journal of Philosophy 1979 76 5 256 282 10.2307/2025881

[B44] Sen A Development as freedom 1999 Oxford: Oxford University Press

[B45] Buchanan A Brock D Daniels N Wikler D From chance to choice. Genetics and justice 2002 Cambridge, Mass.: Oxford University Press

[B46] LeGrand J Equity and Choice. An Essay in Economics and Applied Philosophy 1991 London: Harper Collins Academic

[B47] Cappelen A Norheim O Responsibility in health care: a liberal egalitarian approach Journal of medical ethics 2005 31 8 476 480 10.1136/jme.2004.010421 16076974 PMC1734208

[B48] Cappelen AW Norheim OF Responsibility, fairness and rationing in health care Health Policy 2006 76 312 319 10.1016/j.healthpol.2005.06.013 16112248

[B49] Starfield B State of the art in research on equity in health Journal of Health Politics, Policy and Law 2006 31 1 10.1215/03616878-31-1-11 16484666

[B50] Anand S Sen A Office HDR Human Development Index: Methodology and Measurement Background papers 1994 Washington: Human Development Report Office

[B51] Sen AK Commodities and Capabilities 1985 Amsterdam: North-Holland

[B52] Murray C Lopez A eds The Global Burden of Disease 1996 Cambridge Mass.: Harvard School of Public Health, WHO, World Bank

[B53] Anand S Diderichsen F Evans T Shkolnikov VM Wirth M Evans T, Whitehead M, Diderichsen F, Bhuiya A, Wirth M Measuring disparities in health: methods and indicators Challenging inequities in health: from ethics to action 2001 Oxford: Oxford University Press 49 67

[B54] Daniels N Sabin J Setting limits fairly. Can we learn to share scarce medical resources? 2002 Cambridge: Oxford University Press

[B55] Fleurbaey M Health, wealth and fairness Journal of Public Economic Theory 2005 7 253 284 10.1111/j.1467-9779.2005.00203.x

[B56] Broome J Bell M Mendus S Goodness, fairness and QALYs Philosophy and medical welfare 1988 Cambridge: Cambridge University Press 57 73

[B57] Prah-Ruger J Ethics of the social determinants of health Lancet 2004 364 9439 1092 1097 10.1016/S0140-6736(04)17067-0 15380971 PMC3988689

[B58] Alkire S Chen L Global health and moral values Lancet 2004 364 9439 1069 1074 10.1016/S0140-6736(04)17063-3 15380967 PMC7123313

[B59] Anand P Dolan P Equity, capabilities and health Soc Sci Med 2005 60 2 219 222 10.1016/j.socscimed.2004.04.031 15522479

[B60] Alkire S Why the Capability Approach? Journal of Human Development 2005 6 1 115 133 10.1080/146498805200034275

